# Impact of Exhaled Breath Acetone in the Prognosis of Patients with Heart Failure with Reduced Ejection Fraction (HFrEF). One Year of Clinical Follow-up

**DOI:** 10.1371/journal.pone.0168790

**Published:** 2016-12-28

**Authors:** Fabiana G. Marcondes-Braga, Guilherme L. Batista, Ivano G. R. Gutz, Paulo H. N. Saldiva, Sandrigo Mangini, Victor S. Issa, Silvia M. Ayub-Ferreira, Edimar A. Bocchi, Alexandre Costa Pereira, Fernando Bacal

**Affiliations:** 1 Department of Heart Transplant, Heart Institute (InCor), do Hospital das Clínicas da Faculdade de Medicina da Universidade de São Paulo, Sao Paulo, Brazil Av. Dr Eneas de Carvalho Aguiar, 44 – 2°. andar; 2 Chemistry Institute, University of São Paulo, Sao Paulo, Brazil Av.Prof. Lineu Prestes, 748, bloco 12, sala 1270—Cidade Universitária; 3 Department of Pathology, Faculdade de Medicina da Universidade de São Paulo, Sao Paulo, Brazil Av. Dr. Arnaldo, 455, 1° andar; 4 Department of Heart Failure, Heart Institute (InCor), do Hospital das Clínicas da Faculdade de Medicina da Universidade de São Paulo, Sao Paulo, Brazil; 5 Laboratory of Genetics and Molecular Cardiology, Faculdade de Medicina da Universidade de Sao Paulo, Sao Paulo, Brazil Av.Dr. Eneas de Carvalho Aguiar, 44–10°. andar; The Pennsylvania State University, UNITED STATES

## Abstract

**Background:**

The identification of new biomarkers of heart failure (HF) could help in its treatment. Previously, our group studied 89 patients with HF and showed that exhaled breath acetone (EBA) is a new noninvasive biomarker of HF diagnosis. However, there is no data about the relevance of EBA as a biomarker of prognosis.

**Objectives:**

To evaluate whether EBA could give prognostic information in patients with heart failure with reduced ejection fraction (HFrEF).

**Methods:**

After breath collection and analysis by gas chromatography-mass spectrometry and by spectrophotometry, the 89 patients referred before were followed by one year. Study physicians, blind to the results of cardiac biomarker testing, ascertained vital status of each study participant at 12 months.

**Results:**

The composite endpoint death and heart transplantation (HT) were observed in 35 patients (39.3%): 29 patients (32.6%) died and 6 (6.7%) were submitted to HT within 12 months after study enrollment. High levels of EBA (≥3.7μg/L, 50^th^ percentile) were associated with a progressively worse prognosis in 12-month follow-up (log-rank = 11.06, p = 0.001). Concentrations of EBA above 3.7μg/L increased the risk of death or HT in 3.26 times (HR = 3.26, 95%CI = 1.56–6.80, p = 0.002) within 12 months. In a multivariable cox regression model, the independent predictors of all-cause mortality were systolic blood pressure, respiratory rate and EBA levels.

**Conclusions:**

High EBA levels could be associated to poor prognosis in HFrEF patients.

## Introduction

There is a great number of admissions and re-admissions in hospitals nowadays related to poor prognosis of heart failure (HF).[1,2] Heart failure (HF) is often associated with poor prognosis and frequent hospital admissions and re-admissions. Prognostic markers that allow risk stratification of HF patients may be used to guide medical decision-making. Different types of HF severity and prognosis biomarkers of HF have emerged recently. Among these biomarkers, B-type natriuretic peptide (BNP) is the most studied one[3,4] and it seems to be a good predictor of long-term mortality in patients with chronic HF[5] and acute HF[6].

The mixture of volatile organic compounds present in exhaled breath may be used to diagnose and monitor the disease, advantageously substituting old methods, as it is noninvasive and safe. There could also be potential applications for other cardiovascular diseases.[7]

In a previous study, involving 89 patients with HF enrolled from May 2009 to September 2010, our group showed that levels of acetone are higher in HF patients in comparison to healthy subjects, especially in patients with acute decompensated heart failure (ADHF). According to the authors’ conclusions “*exhaled breath acetone (EBA) seems to be not only a promising non-invasive diagnostic method of HF*, *whose accuracy is equivalent to BNP*, *but also a new biomarker of HF severity*, *since EBA levels differed significantly as a function of severity of HF (NYHA—New York Heart Association classification)”*.[8] Other studies have shown higher levels of acetone in exhaled breath of patients with heart disease[9,10]. However, there is no data on the role of acetone as a prognostic biomarker of HF.

So, we followed these patients for one year to test whether EBA levels in patients with HF could give prognostic information over 12-month follow-up.

## Methods

The 89 patients included in the previous study had left ventricular systolic dysfunction (ejection fraction of no more than 40%) and symptoms of HF according to Framingham criteria[11]. Patient’s characteristics are available in S1 Table. According to the classification of HF by hemodynamic profiles[12], 34% were in profile A, clinically stable in the previous 3 months and the other 66% of patients were admitted to emergency department in profile B, wet and warm (34%) and in profile C, wet and cold (32%). Patients with diabetes, chronic renal failure (creatinine > 2.5 mg/dL or urea > 100mg/dL), chronic hepatic failure and pregnant women were excluded because these comorbidities could interfere in the ketoacid metabolism. Once the written informed consent was obtained from an eligible patient, they were submitted to breath collection using a previously described portable noninvasive device[8,13]^,^ whose collection is detailed in S1 Fig. Breath samples were analyzed by gas chromatography-mass spectrometry (GC-MS) for identification of chemical compounds and by spectrophotometry, after reaction with salicylaldehyde[14] for quantitative analysis. In order to avoid fasting-induced ketone body increase, patients received food at least 60 minutes before sampling.

For the purposes of the current study, patients were followed by one year after collection. The composite endpoint of the study was death or heart transplantation in one year. Study physicians, who ascertained vital status of each study participant at 12 months, adjudicated it. Physicians obtaining follow-up data were blind to the results of cardiac biomarker testing. Institutional Ethics Committee (*CAPPesq—Comissão de Ética para Análise de Projetos de Pesquisa da Diretoria Clínica do Hospital das Clínicas e da Faculdade de Medicina da Universidade de São Paulo*) approved the study (1045/07).

### Statistical analysis

The SPSS (*Software Statistical Package for the Social Science*) was used for statistical analysis. Categorical variables were shown as absolute (n) and relative frequency (%). To test normality we used the Kolmogorov-Smirnov test. Parametric data were shown as mean and standard deviation. Non-parametric data were shown as median and lower (25^th^ percentile) and upper quartile (75^th^ percentile). Univariable survival curves (Kaplan Meier) were computed to assess the influence of acetone in survival rate. We also used univariable and multivariable cox proportional regression model to predict mortality or heart transplantation in 12 months.

## Results

Table 1 depicts clinical data of HF patients. The median age was 62 years and 62% of patients were male. The main etiology of cardiomyopathy was *Chagas* disease (33%), followed by idiopathic (23%), hypertensive (18%) and ischemic (13%) cardiomyopathy. Most of patients were NYHA Functional Class 3 or 4 (78%). Median left ventricular ejection fraction was 24%, median left ventricle diastolic diameter was 67 mm and lab tests revealed median serum creatinine of 1.22 (0.97–1.80) mg/dL, serum urea of 51 (37–74) mg/dL and median EBA of 3.70 (1.69–10.45 μg/L). Ninety-four percent of patients were receiving angiotensin converting enzyme inhibitor or angiotensin II receptor blocker; 100% betablocker; 69% spironolactone and 81% loop diuretics.

**Table 1:** Baseline Characteristics of patients with Heart Failure.

Characteristics	HF patients (n = 89)
Male n(%)	55 (62)
Age (years)	62.0 (44.5–61.0)
Weight (g)	65.0 (59.5–76.0)
Body Mass Index	24.0 (21.8–26.5)
Race n(%)	
White	51 (57)
Black	38 (43)
Etiology n(%)	
Ischemic	12 (13)
Chagas	33 (37)
Hypertensive	16 (18)
Idiopathic	20 (23)
Other	8 (9)
NYHA n(%)	
1/2	20 (22)
3	39 (44)
4	30 (34)
Hemodynamic profile n(%)	
A	30 (34)
B	30 (34)
C	29 (32)
History n(%)	
Hypertension	34 (38)
Dyslipidemia	24 (27)
Myocardial Infarction	16 (18)
Stroke	11 (13)
Smoking	2 (2)
Atrial fibrillation (%)	19 (21)
Pace maker/ IDC (%)	14 (16)
Physical examination	
SBP (mmHg)	96 (80–110)
DBP (mmHg)	70 (60–70)
Heart rate (bpm)	68 (60–76)
Respiratory rate (irm)	22 (18–28)
Echocardiography	
LVEF (%)	24.0 (19.5–30)
LVDD (mm)	67.0 (62.0–73.5)
Diastolic Dysfunction	42 (47)
RV Dysfunction	55 (62)
Laboratory	
Hemoglobin (g/dl)	13.0 (12.1–14.1)
Urea (mg/dl)	51.0 (37.0–74.0)
Creatinine (mg/dl)	1.22 (0.97–1.80)
Sodium (mEq/L)	138 (136–140)
Potassium (mg/dl)	4.4 (4.0–4.8)
Glucose (mg%)	92.0 (85.0–99.0)
EBA (μg/L)	3.70 (1.69–10.45)
Drugs	
ACEI	71 (80)
ARB	13 (15)
ACEI/ARB	84 (94)
β-blocker	89 (100)
Spironolactone	61 (69)
Loop diuretic	72 (81)
Thiazide diuretic	17 (19)
Hydralazine/Nitrate	33 (37)
Digoxin	33 (37)
Amiodarone	12 (14)

Continuous values were expressed in median (25^th^ percentile– 75^th^ percentile).

IDC–implantable defibrillator cardioverser; A–hot and dry; B–hot and wet;

C–cold and wet; SBP–systolic blood pressure; DBP–diastolic blood pressure;

LVEF–left ventricular ejection fraction; LVDD–left ventricle diastolic diameter;

RV–right ventricle; ACEI-angiotensin converting enzyme inhibitor;

ARB–angiotensin II receptor blocker.

### Exhaled acetone as a predictor of mortality or heart transplantation in 12 months

Considering the severity of the disease, a great number of patients with HF is considered to heart transplantation or left ventricular device use. The composite endpoint death or heart transplantation was observed in 35 out of 89 patients of this population (39.3%): 29 (32.6%) deaths and 6 (6.7%) heart transplants within 12 months after study enrollment. No patient was submitted to left ventricular assistance device.

The great majority of patients died due to HF progression (17 patients, 58,6%); three died of infection; four had a sudden death; four other died at home of unknown cause and one died during the heart transplant surgery.

We have studied the role of acetone as a predictor of 12-month mortality or heart transplantation in this group of HF patients. By cox regression, we have shown that EBA could predict mortality or heart transplantation in 12 months in this group of HF patients (HR = 1.05, 95%CI = 1.02–1.08, p = 0.002) (Table 2).

**Table 2:** Univariable Cox Proportional Regression Analysis for 12-month mortality or heart transplantation.

Variables	HR	95%CI	p
EBA>3.7ug/L	3.26	1.56–6.80	0.002
EBA (continuous variable)	1.05	1.02–1.08	0.002
Creatinine	2.49	1.48–4.16	0.001
Urea	1.01	1.00–1.01	0.006
BNP	1.003	1.001–1.005	0.002
NYHA class I/II			0.002
class III	2.35	0.67–8.25	
class IV	6.04	1.79–20.47	
Age	1.02	0.99–1.04	0.302
SBP	0.97	0.95–0.99	0.003
BMI	0.91	0.84–0.99	0.031
Sex	1.00	0.51–1.99	0.992
Respiratory rate	1.10	1.04–1.16	0.001
Heart rate	0.99	0.96–1.02	0.384
LVEF	0.96	0.92–1.01	0.104
Atrial fibrillation	0.98	0.44–2.16	0.964
Myocardial infarction	1.11	0.49–2.55	0.800
Hemoglobin	0.84	0.67–1.06	0.147
Sodium	0.93	0.86–1.02	0.108
Lactate	1.02	0.98–1.06	0.380
Medication			
ACEI	0.99	0.43–2.28	0.989
Spironolactone	0.90	0.45–1.81	0.763
Furosemide	2.18	0.77–6.17	0.144
Digoxin	1.14	0.58–2.24	0.704
Hydralazine	0.81	0.39–1.69	0.572
Nitrate	1.89	0.93–3.86	0.081
Amiodarone	1.46	0.61–3.53	0.399

Beta-blocker was not included in the analysis because 100% of patients were taking this medicine.

HR: hazard ratio; EBA: exhaled breath acetone; SBP: systolic blood pressure; BMI: body mass index; LVFE: left ventricular ejection fraction; BNP: B-type natriuretic peptide (each 10 units)

In order to give practical application to this new biomarker, we have stratified EBA levels according to 50^th^ percentile (median) value and evaluated its role in predicting mortality or heart transplantation in HF patients. We have shown that a concentration of EBA higher than 3.7 μg/l (50^th^ percentile) increased the risk of death or heart transplantation within 12 months in 3.3 times (HR = 3.26, 95%CI = 1.53–6.80, p = 0.002) (Table 2). EBA ≥ 3.7 μg/l was able to predict 12-month mortality or heart transplantation in HF patients with New York Heart Association (NYHA) 3/4 (HR = 2.57, 95%CI = 1.11–5.96, p = 0.028), but not in NYHA 1/2 patients.

All variables considered potential predictors of mortality in HF patients (age, sex, body mass index, systolic blood pressure, rhythm, heart rate, ejection fraction, NYHA class, etiology, urea, creatinine, sodium, hemoglobin, B-type natriuretic peptide (BNP), lactate and HF medications) were included in univariable COX regression model. To be included in multivariable COX regression model, variables should have p < 0.10 in univariable model analysis. The independent predictors of all-cause 12-month mortality or heart transplantation were EBA>3.70ug/L (HR = 2.32, 95%CI = 1.10–4.92, p = 0.028) and respiratory rate (HR = 1.09, 95%CI = 1.03–1.18, p = 0.006). Systolic blood pressure was associated with lower 12-month mortality or heart transplantation (HR = 0.98, 95%CI = 0.96–0.99, p = 0.007). Table 3 depicts the multivariable Cox proportional regression analysis for the composite endpoint considering EBA as a categorical and a continuous variable.

**Table 3:** Multivariable Cox Proportional Regression Analysis for 12-month mortality or heart transplantation.

**Considering EBA as a continuous variable**			
**Variables**	**HR**	**95%CI**	**p**
Respiratory rate	1.10	1.04–1.17	0.002
EBA	1.04	1.001–1.071	0.043
SBP	0.98	0.96–0.99	0.010
**Considering EBA categorized by median value (3.7 ug/L)**			
**Variables**	**HR**	**95%CI**	**p**
EBA > 3.70 ug/L	2.32	1.10–4.92	0.028
Respiratory rate	1.09	1.03–1.17	0.006
SBP	0.98	0.96–0.99	0.007

EBA: exhaled breath acetone; SBP: systolic blood pressure; HR: hazard ratio.

Variables included in multivariable analysis: systolic blood pressure (SBP); age; body mass index (BMI); respiratory rate; NYHA class; creatinine; urea; BNP (B-type natriuretic peptide) and exhaled breath acetone (EBA)

We have also analyzed heart transplantation or mortality rate according to 50^th^ percentile (median) of exhaled acetone. In 12 months, among patients with EBA ≥ 3.7μg/L (50^th^ percentile–median of exhaled acetone), there were 21 deaths and 4 patients were submitted to heart transplant. Among patients with EBA < 3.7μg/L there were 8 deaths and 2 heart transplants.

**Fig 1:**
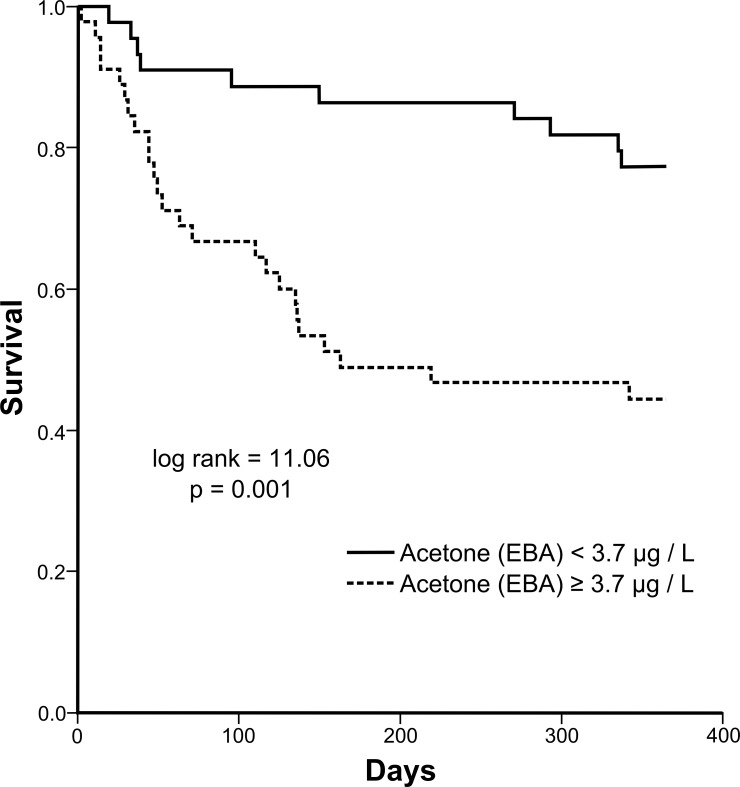
Kaplan Meier survival curves for acetone stratified above and below the median value against all-cause mortality or heart transplantation in 12 months (log rank test, p = 0.001).

Survival curves for EBA stratified above and below the median value against all-cause mortality or heart transplantation in 12 months are shown below in Fig 1. Concentrations of EBA above 3.7μg/L were associated with a progressively worse prognosis in 12-month follow-up (log-rank = 11.06, p = 0.001).

## Discussion

Different studies have demonstrated that acetone is increased in exhaled breath of patients with HF when compared to healthy subjects.[8,10,15] Considering that levels of EBA increase in accordance with NYHA classification, it should be highlighted that EBA can also be used as a new biomarker of HF severity.[8] The current study suggests that EBA could be also a good predictor of long-term mortality or heart transplantation in HF patients, even when other well-known predictors of mortality are considered.

It is known that plasma catecholamines are increased in patients with HF. Plasma free fatty acids concentrations increase with lipolysis induced by the high levels of norepinephrine. This might occur due to greater β-adrenergic stimulation.[16] A series of condensation reactions that produce tree types of ketone bodies (namely acetoacetate, β-hydroxybutyrate and acetone) happen in advanced HF. This results from an increase in acetyl-CoA levels in mitochondria, caused by high levels of free fatty acids. Acetoacetate and β-hydroxybutyrate are metabolically active, but acetone provides a convenient volatile odor for detection of ketosis. Liver enzymes are able to produce ketone bodies, but are not able to metabolize them. In this way, under glucose scarcity conditions, there is an accumulation of ketone bodies in the blood in order to provide energy for tissues in the heart, brain, kidney and muscles. In this situation, acetone can be detected in exhaled breath.[17]

It is well established that BNP is a good prognostic biomarker of HF. In chronic HF, high levels of BNP are associated with higher mortality and morbidity in different studies[5,18] and this biomarker could be a good predictor of sudden death.[19] In acute HF, BNP seems to predict long-term mortality when in high concentration.[6] Natriuretic peptides have been tested as biomarkers of short-term mortality and most of studies showed these peptides could be also good predictors of hospital admissions and death.[20]

Other biomarkers as mid-region pro-adrenomedulin[21]; copeptin[22]; soluble sT2[23]; high sensitivity troponin[24]; galectin-3[25] have been described as biomarkers of HF. Most of them proved to be not only good predictors of mortality in HF patients, but also biomarkers that add prognostic information when associated to natriuretic peptides.

However, a biomarker that could simultaneously reveal mechanisms of physiopathology of the disease and allow diagnostic and prognostic evaluation has not been described yet. In this context, exhaled breath acetone could be considered a potential biomarker of HF, since it reflects metabolic changes of advanced HF.

In this current study, high levels of EBA were associated to higher 12-month mortality or heart transplantation in HF patients. When other well-known prognostic biomarkers such as NYHA class, urea, creatinine, sodium, hemoglobin, systolic blood pressure and BNP were considered, EBA proved to be an independent predictor of long-term mortality or heart transplantation in this population.

### Study limitations

One apparent limitation of this study is the small sample size (89 HF patients, 35 events). Since there is no previous data on this topic, it was not possible to calculate the appropriated sample size for this study. So, we have followed HF patients included in a previous study by one year[8]. Another study limitation is that in this first research, we studied patients in different hemodynamic profiles (34% with chronic HF and 66% with acute decompensated HF), which gives some heterogeneity to the sample. Nevertheless, available results on the potential of EBA as a new biomarker for long-term mortality/heart transplantation prediction are relevant and might inspire prompt confirmatory larger studies.

## Conclusions

The current study suggests that high EBA levels could be associated with high mortality or heart transplantation in HF patients. However, considering the small size, we know that this is a hypothesis generation study. Regarding the interesting results of this study, larger prospective trials should be drawn to confirm the role of acetone as a long-term prognostic biomarker of chronic and acute HF.

## Supporting Information

S1 Table Baseline Characteristics of heart failure patients. This is the S1 Table legend: Continuous values were expressed in median (interquartile range). HF–heart failure; SBP–systolic blood pressure; DBP–diastolic blood pressure; LVEF–left ventricular ejection fraction; LVDD–left ventricle diastolic diameter.
(DOC)

S1 Fig Collector device and collection of exhaled breath. This is the S1 Fig legend: disposable inlet tube (A); glass bubbler (impinger) (B); diffuser (C); distilled icy water (D); ice/water bath (E); empty plastic bag (F).
(DOC)
