# Supplementary material for: Impact of Exhaled Breath Acetone in the Prognosis of Patients with Heart Failure with Reduced Ejection Fraction (HFrEF). One Year of Clinical Follow-up
Source: PLoS One. 2016 Dec 28;11(12):e0168790. doi: 10.1371/journal.pone.0168790 (PMC5193433; doi:10.1371/journal.pone.0168790)
Supplement: S1 Table — This is the S1 Table legend: Continuous values were expressed in median (interquartile range). HF–heart failure; SBP–systolic blood pressure; DBP–diastolic blood pressure; LVEF–left ventricular ejection fraction; LVDD–left ventricle diastolic diameter. (DOC) [file pone.0168790.s001.doc]

**Supporting information file**

**S1 Table**

**Table – Baseline Characteristics of heart failure** patients

| **Characteristics** | **HF (n=89)** |
| --- | --- |
| Male n(%) | 55 (62.0) |
| Age (years) | 52.0 (44.5 – 61.0) |
| Weight (kg) | 65.0 (59.5 – 76.0) |
| White race n(%) | 51 (57) |
| History n(%) |  |
| Hypertension | 34 (38) |
| Dyslipidemia | 24 (27) |
| Myocardial Infarction | 16 (18) |
| Stroke | 11 (13) |
| Smoking | 2 (2) |
| Physical exam |  |
| SBP (mmHg) | 96 (80 – 110) |
| DBP (mmHg) | 70 (60 – 70) |
| Heart rate (bpm) | 68 (60 – 76) |
| Respiratory rate (irm) | 22 (18 – 28) |
| O_2_ Saturation (%) | 95.0 (94.0 – 96.0) |
| Echocardiography |  |
| LVEF (%) | 24.0 (19.5 – 30.0) |
| LVDD (mm) | 67.0 (62.0 – 73.5) |
| Laboratory |  |
| Hemoglobin (g/dl) | 13.1 (12.1 – 14.3) |
| Urea (mg/dl) | 51.0 (37.0 – 74.0) |
| Creatinine (mg/dl) | 1.22 (0.97 – 1.80) |
| Sodium (mEq/L) | 138 (136 – 140) |
| Potassium (mEq/L) | 4.4 (4.0 – 4.8) |
| Glucose (mg%) | 92.0 (85.0 – 99.0) |

Continuous values were expressed in median (interquartile range).

HF – heart failure; SBP – systolic blood pressure; DBP – diastolic blood pressure;

LVEF – left ventricular ejection fraction; LVDD – left ventricle diastolic diameter.
