# Supplementary material for: Impact of Exhaled Breath Acetone in the Prognosis of Patients with Heart Failure with Reduced Ejection Fraction (HFrEF). One Year of Clinical Follow-up
Source: PLoS One. 2016 Dec 28;11(12):e0168790. doi: 10.1371/journal.pone.0168790 (PMC5193433; doi:10.1371/journal.pone.0168790)
Supplement: S1 Fig — This is the S1 Fig legend: disposable inlet tube (A); glass bubbler (impinger) (B); diffuser (C); distilled icy water (D); ice/water bath (E); empty plastic bag (F). (DOC) [file pone.0168790.s002.doc]

**S2 Fig**

**Collector device and collection of exhaled breath**

A simple and portable breath collector was developed for this study. During the collection, the patient blew through a disposable inlet tube (A) to a glass bubbler (impinger) (B), which contained 5.0 ml of distilled icy water (D) and was immersed in an ice/water bath (E). The generation of small bubbles in cold water by a diffuser (C) increased gas/water contact and enhanced the efficiency of soluble compounds extraction. A short hose of an otherwise sealed empty plastic bag (F) was connected to the impinger output duct, enabling the collection of a fixed volume of breath (7.6 liters) from each patient. The patients were asked to breathe several times into the collector, consecutively, until they had filled the bag with 7.6 liters of air, which allowed collection of alveolar air. After collection, the sample was kept under cooled conditions -112°F(-80^o^C) and analyzed by gas chromatography-mass spectrometry (GC-MS) for identification of chemical compounds and by spectrophotometry, after reaction with salicylaldehyde for quantitative analysis. Patients received food at least 60 minutes before sampling to avoid fasting-induced ketone body increase.

**
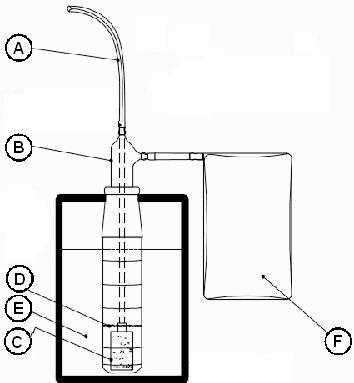
**
